# A High Crystalline Perylene-Based Hydrogen-Bonded Organic Framework for Enhanced Photocatalytic H_2_O_2_ Evolution

**DOI:** 10.3390/molecules28196850

**Published:** 2023-09-28

**Authors:** Mengke Hu, Chenxi Wu, Shufan Feng, Jianli Hua

**Affiliations:** Key Laboratory for Advanced Materials, School of Chemistry and Molecular Engineering, East China University of Science and Technology, 130 Meilong Road, Shanghai 200237, China; y30210401@mail.ecust.edu.cn (M.H.); y30230414@mail.ecust.edu.cn (C.W.); y12223040@mail.ecust.edu.cn (S.F.)

**Keywords:** photocatalysis, hydrogen peroxide, perylene, hydrogen-bonded organic framework

## Abstract

Hydrogen-bonded organic frameworks (HOFs) are a kind of crystalline porous material that have shown great potential for photocatalysis on account of their mild synthesis conditions and high crystallinity. Perylene-based photocatalysts have great potential for photocatalytic H_2_O_2_ production due to their excellent photochemical stability and broad spectral absorption. In this work, we designed and synthesized a high crystalline perylene-based HOF (PTBA) and an amorphous analog sample PTPA for photocatalytic H_2_O_2_ evolution. Under visible light irradiation, PTBA shows a higher photocatalytic H_2_O_2_ production rate of 2699 μmol g^−1^ h^−1^ than PTPA (2176 μmol g^−1^ h^−1^) and an apparent quantum yield (AQY) of 2.96% at 500 nm. The enhanced photocatalytic performance of PTBA is attributed to the promotion of the separation and transfer of photocarriers due to its high crystallinity. This work provides a precedent for the application of HOFs in the field of photocatalytic H_2_O_2_ generation.

## 1. Introduction

As an environmentally friendly versatile oxidant, hydrogen peroxide (H_2_O_2_) is widely used in water treatment [1], antibacterial material [2], chemical synthesis [3], etc. Industrial hydrogen peroxide is produced mainly through anthraquinone (AQ) oxidation [4]. On account of the high energy consumption and hazardous solvent waste generation of the AQ oxidation process, alternative environmentally friendly methods for H_2_O_2_ production are highly desired [5,6]. Contrasted with traditional strategies, photocatalytic H_2_O_2_ generation is a promising approach that uses water, oxygen, and sunlight as sources, which involves no substantial environmentally harmful or serious safety issues [7,8,9,10]. Semiconductor photocatalysts play a crucial part in photocatalysis [11], among which organic photocatalysts have shown excellent light absorption ability [12] as well as appealing structural and functional diversity [13,14]. Due to its excellent photochemical stability and broad spectral absorption, perylene has been found to be a promising photocatalyst in effective photocatalytic H_2_O_2_ evolution. For instance, Hua et al. [15] prepared a series of type II heterojunctions by combining the bay-annulated perylene imides with g-C_3_N_4_, in which these heterojunctions exhibited a high photocatalytic H_2_O_2_ evolution rate. The key to the enhanced performance is the increased discrepancy of electron distribution induced by the introduction of hybrid atom substituents at the bay position. Xiao et al. [16] constructed a Z-scheme heterojunction using a perylene diimide organic supermolecule as a component. The photocatalyst was used for the environmentally friendly removal of organic contaminants. Xu et al. [17] developed a 3D/1D heterostructure using supramolecular perylene diimide as the 1D material. The photocatalyst performed well on disinfection, pollutant degradation, and H_2_O_2_ production. All these investigations have demonstrated the great potential of perylene-based photocatalysts for photocatalytic H_2_O_2_ production. Nonetheless, structural disorders or other extrinsic sources in organic materials could play the role of recombination centers, resulting in reduced efficiency of photogenerated charge carrier migration [18,19]. Various materials have been developed to overcome these limitations. For instance, conjugated organic frameworks (COFs) have been constructed to deeply explore the relationship between structure and function [20,21]. However, the strict synthesis conditions of COFs limit their development in simple and effective photocatalysis [22].

Self-assembled through hydrogen bonding, the hydrogen-bonded organic frameworks (HOFs), whose concept was established in the early 1990s [23], have recently drawn significant attention [24]. In terms of bonding energies, the hydrogen bonds in HOFs are significantly more adaptable and more flexible than covalent bonds in COFs [25], providing HOFs with mild synthetic conditions [26], easy recyclability [27], and well-defined structures, as well as versatility [28,29]. The high crystallinity in HOFs would narrow the band gap and enhance the light-harvesting efficiency, thus accelerating the rapid transport of carriers to improve carrier mobility and conductivity [20]. However, few HOFs were reported for photocatalytic H_2_O_2_ generation. The design of highly active HOFs combined with excellent photostability for photocatalytic H_2_O_2_ generation applications remains a big challenge.

**Scheme 1:**
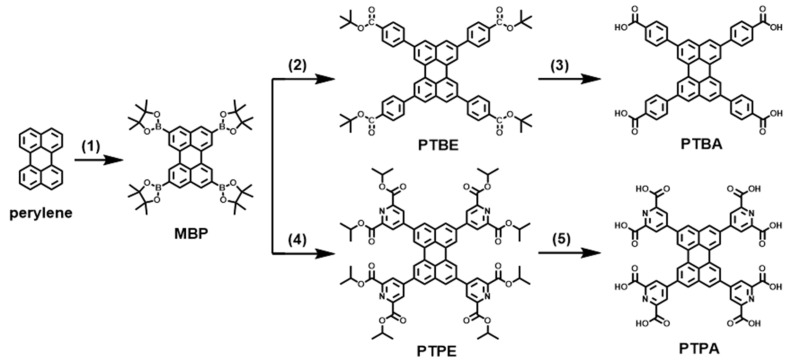
Synthesis of PTPA and PTBA. Reagents and conditions: (1) [Ir(OMe)COD]_2_, 4,4-Dimethyl-2,2′-bipyridyl, 4,4,4′,4′,5,5,5′,5′-octamethyl-2,2′-bi(1,3,2-dioxaborolane), 80 °C, 17 h, (2) tert-butyl 4-iodobenzoate, Pd(dppf)Cl_2_, K_3_PO_4_, 70 °C, 4 h, (3) NaOH, 80 °C, overnight, HCl, (4) diisopropyl 4-iodopyridine-2,6-dicarboxylate, Pd(dppf)Cl_2_, K_3_PO_4_, 70 °C, 4 h, (5) NaOH, 80 °C, overnight, HCl.

Herein, we report a porous HOF named PTBA for efficient H_2_O_2_ production. The amorphous analog PTPA is also designed and synthesized for comparison (Scheme 1). By connecting perylene with benzoic acid and the formation of intermolecular hydrogen bonds, PTBA shows high crystallinity with simple topology, which leads to the promotion of separation and transfer of photocarriers. Impressively, the photocatalytic H_2_O_2_ production rate of PTBA can reach as high as 2699 μmol g^−1^ h^−1^ under visible light irradiation, over 500 μmol g^−1^ h^−1^ higher than the analog PTPA. Our investigations reveal the enormous potential of HOFs for efficient and stable photocatalytic H_2_O_2_ generation.

## 2. Results and Discussion

### 2.1. Synthesis of Photocatalysts

The synthetic routes of PTPA and PTBA are shown in Scheme 1. The intermediate MBP was obtained through the reported procedure with a slight modification [30]. Then, the important intermediates PTBE and PTPE were synthesized through a Suzuki–Miyaura coupling reaction between MBP and tert-butyl 4-iodobenzoate and diisopropyl 4-iodopyridine-2,6-dicarboxylate, respectively. Eventually, PTBE and PTPE were separately hydrolyzed to obtain PTBA and PTPA. Highly crystalline HOF PTBA and amorphous analog photocatalyst PTPA can be facilely constructed through a self-assembly process in DMF and H_2_O at room temperature, according to reference [31].

### 2.2. Structural Characterization and Morphology Analysis

**Figure 1:**
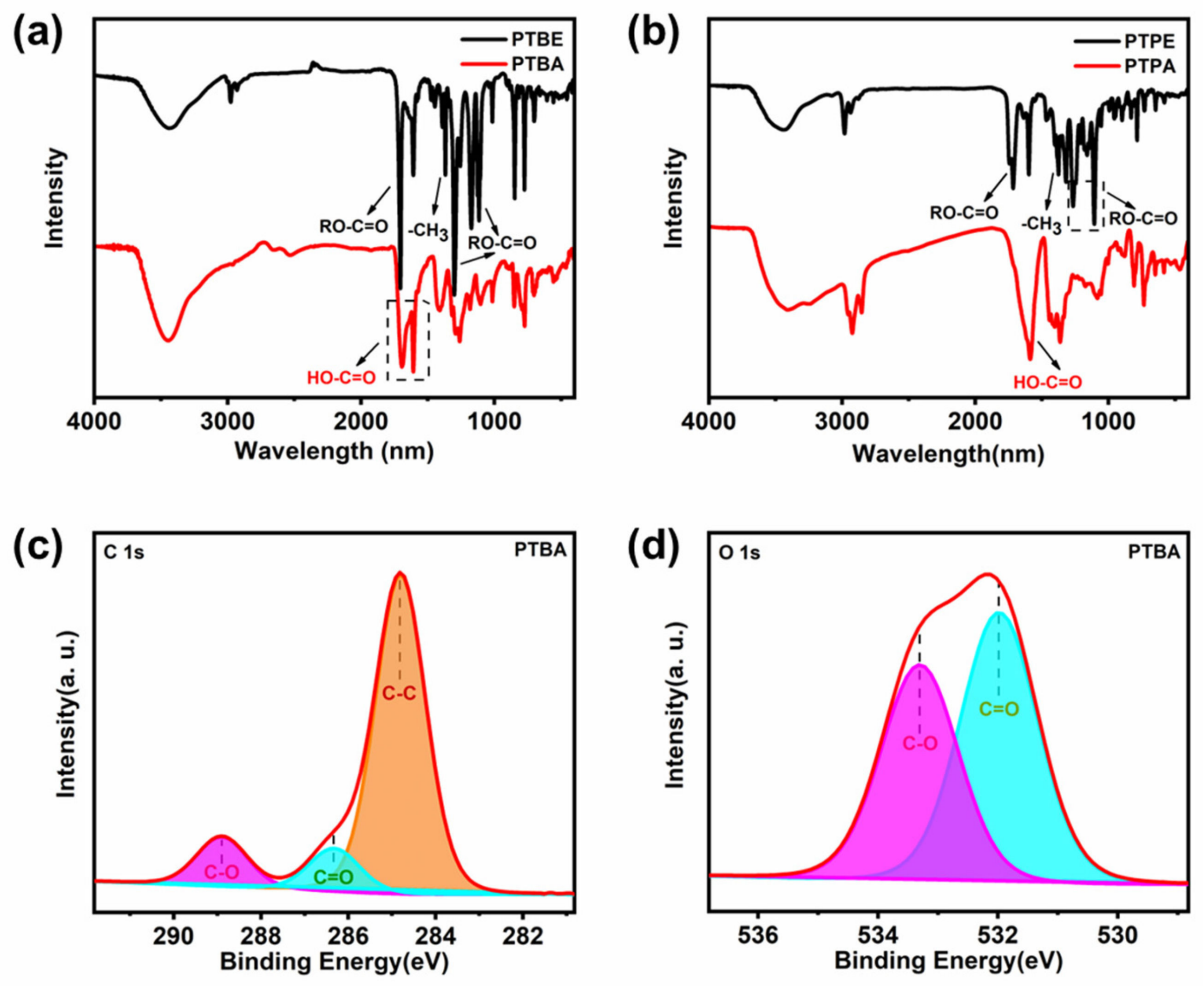
(**a**) FTIR spectra of PTBE and PTBA; (**b**) FTIR spectra of PTPE and PTPA; XPS spectra of PTBA (**c**) C 1s and (**d**) O 1s.

Due to the lack of solubility in common solvents (DCM, THF, CHCl_3_, DMSO, MeOH, EtOH, acetone, and ethyl acetate), the two photocatalysts were characterized by Fourier-transform infrared (FT-IR) spectroscopy and X-ray photoelectron spectroscopy (XPS). The FT-IR spectra show the disappearance of an ester bond C=O stretch vibration at 1702 cm^−1^ in PTBE and the appearance of new peaks at approximately 1691 and 1608 cm^−1^ in PTBA, which are assigned to the C=O of carboxyl-terminal (Figure 1a). Furthermore, a typical methyl bonding vibration at 1366 cm^−1^ in the tertiary butyl groups and two C-O-C stretch vibration peaks at 1300 and 1114 cm^−1^ in the spectrum of PTBE disappear in PTBA, which confirms the successful conversion from ester to carboxylic acid. The results of FTIR spectra demonstrate the successful introduction of carboxyl groups in PTBA, which laid the foundation for the formation of the organic framework through intermolecular hydrogen bonding. Similar vibrations can also be found in the FT-IR spectra of PTPE and PTPA in Figure 1b.

X-ray photoelectron spectroscopy (XPS) has been performed on the samples for structural characterization. In the XPS C1s spectra (Figure 1c), the characteristic peaks at 284.8, 286.33, and 288.88 eV correspond to the C-C, C=O, and O-C=O groups, respectively. As shown in Figure 1d, the O 1s spectra of PTBA and PTPA exhibit two peaks at 531.97 and 533.31 eV, which are separately corresponding to the O=C and O-C bonds [22]. The related groups can also be found in the XPS spectra of PTPA in Figure S1. The results of XPS and FTIR match the characteristics of the proposed structures, which confirms the successful synthesis of PTBA and PTPA.

**Figure 2:**
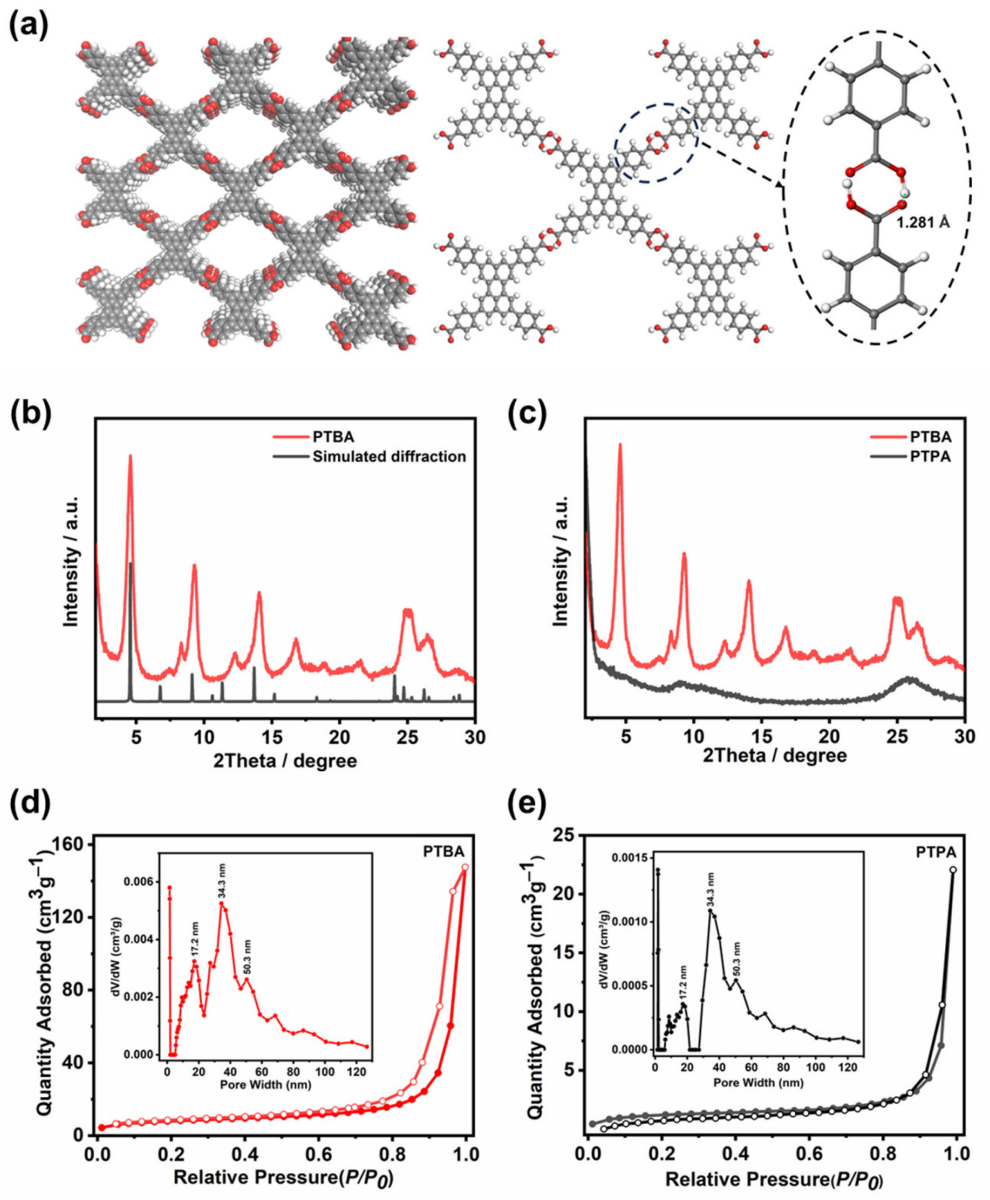
(**a**) The eclipsed structure model of PTBA and a view of the HOF structure and the schematic representation of intermolecular hydrogen bond in PTBA, C (grey), H (white), O (red); (**b**) experimental and simulated XRD patterns of PTBA; (**c**) XRD patterns of PTBA and PTPA; nitrogen adsorption–desorption curve and pore-size distribution (inset) of (**d**) PTBA and (**e**) PTPA, hollow circles: adsorption, filled circles: desorption.

**Figure 3:**
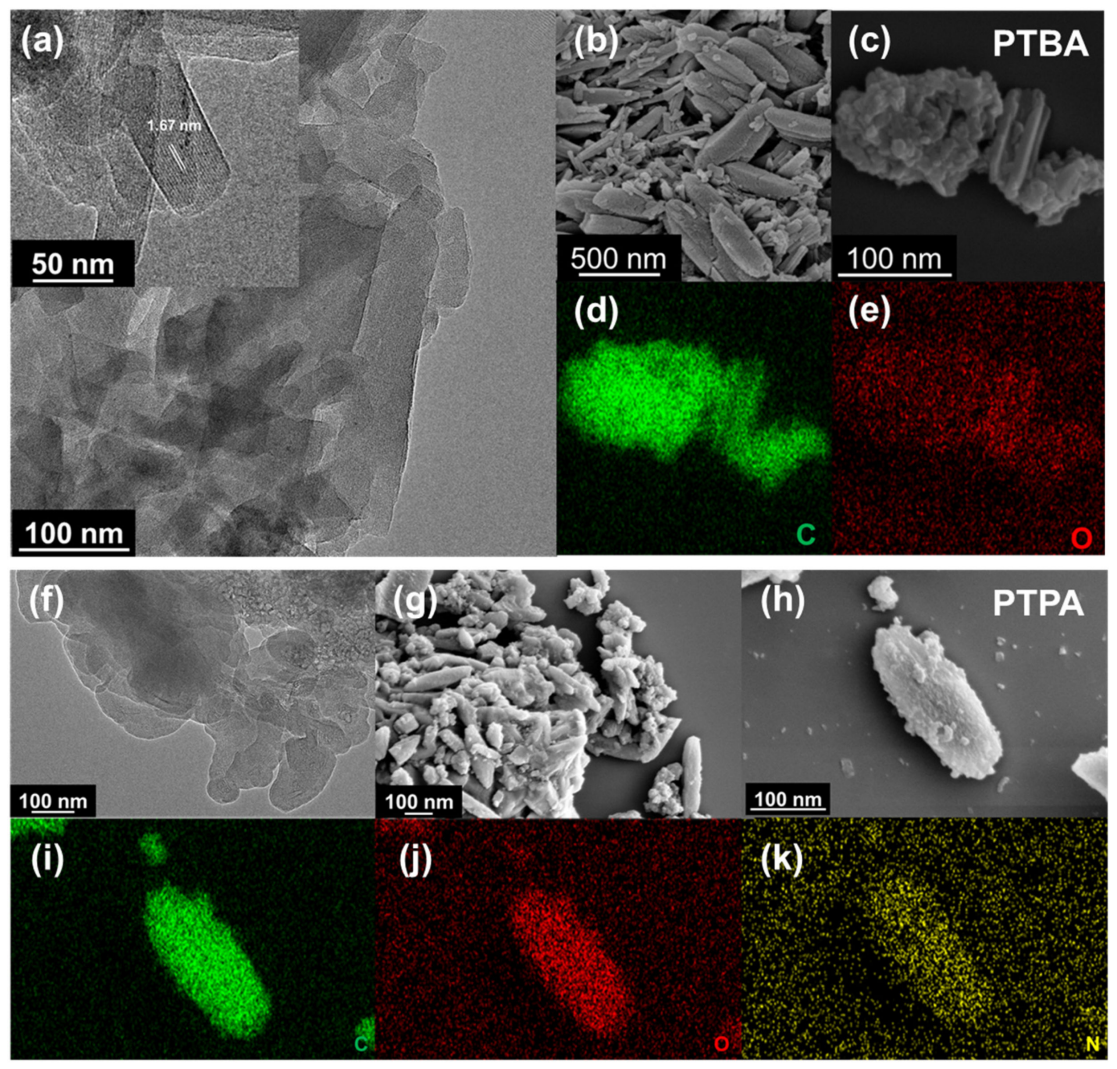
(**a**) TEM, (**b**) SEM images at different view angles, (**c**–**e**) EDS mapping images of PTBA; (**f**) TEM, (**g**) SEM images at different view angles, (**h**–**k**) EDS mapping images of PTPA.

To better understand the structure of HOF PTBA, a simulated molecular model was established. As illustrated in Figure 2a, the distance between the carbonyl oxygen atom of a PTBA molecule and the hydroxyl hydrogen atom of the adjacent molecule is simulated as 1.281 Å, meeting the conditions of hydrogen bond formation, which provides preliminary evidence for the successful synthesis of HOF. The crystalline structure of PTBA was determined by means of powder X-ray diffraction (PXRD) analysis. The sharp diffraction peak at 4.54°, which is assigned to the (110) plane, demonstrates the formation of a framework for PTBA (Figure 2b). However, PTPA shows an amorphous feature. This may be the reason that the introduction of more carboxyl groups reduces the solubility of PTPA in organic solvents, leading to faster precipitation, which is not conducive to crystal formation. (Figure 2c). Additionally, the broad peaks at around 25° in both samples point to the π-π stacking interaction between the molecules [32]. The surface area and porosity of PTBA and PTPA were measured by nitrogen adsorption analysis, and the BET surface area was calculated to be 28.82 m^2^ g^−1^ for PTBA and 4.52 m^2^ g^−1^ for PTPA (Figure 2d,e). PTBA shows a higher surface area than PTPA, arising from its highly ordered structure, which allows for more reactive site exposure. Their pore size distributions are exhibited in the insets in Figure 2d,e, presenting mesoporous structures with pore widths of about 17.2 and 34.3 nm. As illustrated in Figure 3a, the ordered stacked structure of PTBA is verified by the transmission electron microscopy (TEM) images through the lattice fringes with a stripe spacing of 1.67 nm. Scanning electron microscopy (SEM) images (Figure 3b) visually show two kinds of cuboid morphology of PTBA on a nanometer scale, while PTPA represents a spindle-like structure (Figure 3g). The energy-dispersive X-ray spectroscopy (EDS) mapping analysis (Figure 3c–e) reveals that the content of C and O elements is well distributed in PTBA.

### 2.3. Photochemical and Photoelectrochemical Properties

**Figure 4:**
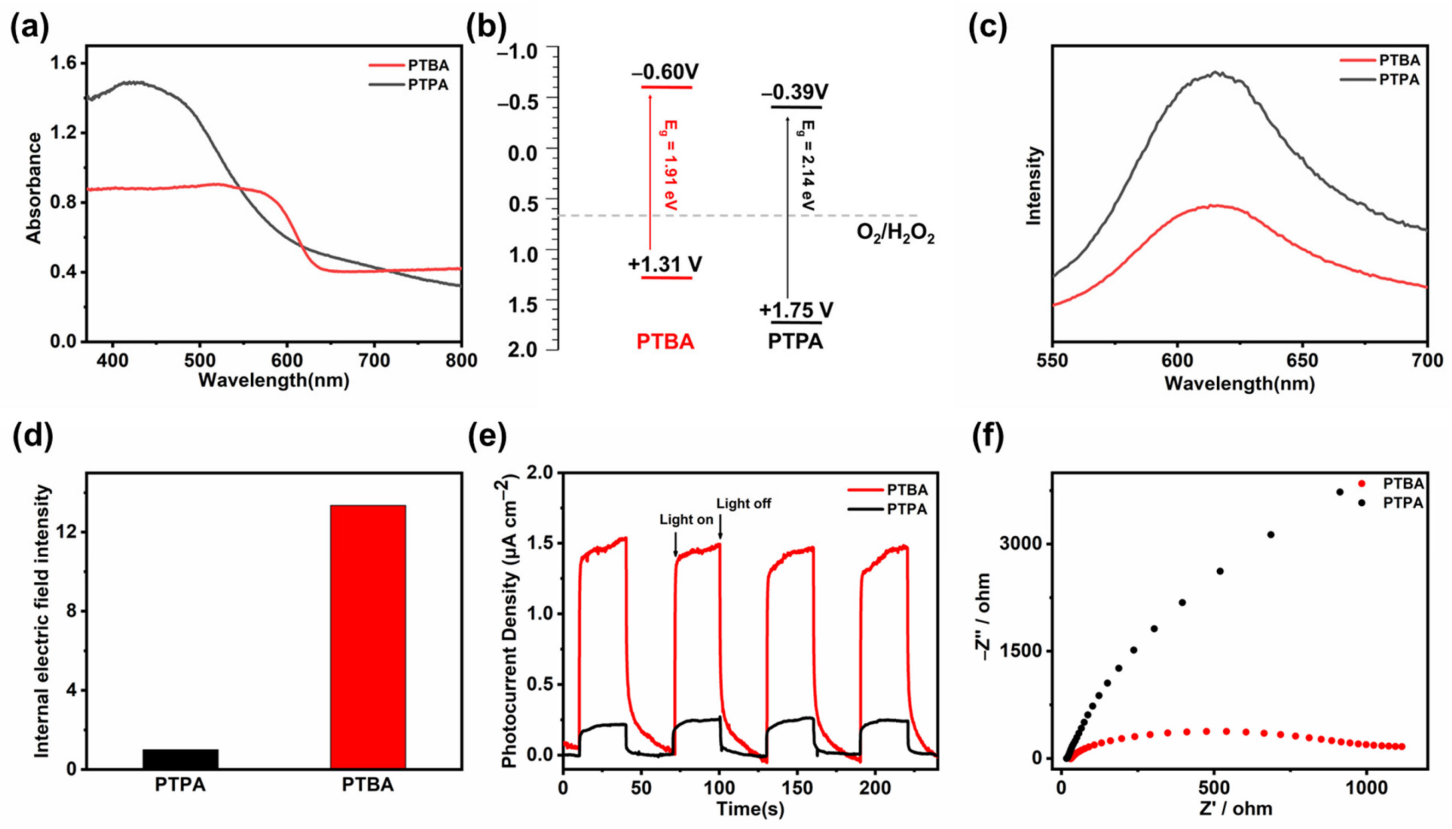
(**a**) UV-vis DRS spectra, (**b**) band diagram, (**c**) PL spectra, (**d**) normalized internal electric field, (**e**) transient photocurrent curves, and (**f**) EIS Nyquist plots of PTBA and PTPA.

As illustrated in Figure 4a, UV-vis diffuse reflectance spectra (DRS) were obtained to investigate the photon capture ability of the samples. Correspondingly, based on the UV-vis absorption results, the band gaps (E_g_) calculated from Tauc-Plots are 1.91 and 2.14 eV for PTBA and PTPA, respectively (Figure S2). In addition, the Mott–Schottky (MS) measurements were conducted to calculate the conduction band (CB) positions of PTBA and PTPA (Figure S3). The positive slope of typical MS plots indicates an n-type feature of the compounds, and the corresponding flat-band potentials (E_fb_) of PTBA and PTPA are fitted to be −0.64 and −0.43 V, respectively. For n-type semiconductors, the CB potential (E_CB_) is 0.2 V more positive compared to the flat-band potential, and the value of ψ (SCE) is 0.24 V more negative under experimental conditions. Consequently, E_CB_ vs. the normal hydrogen electrode (NHE) at pH = 7 can be calculated based on the following formula [33].

E_CB_ (vs. NHE, pH = 7) = E_fb_ (vs. SCE, pH = 7) + 0.24 − 0.2

Therefore, E_VB_ values of PTBA and PTPA are calculated to be −0.6 and −0.39 V, respectively. Notably, even though PTPA has higher absorption in short wavelength regions for capturing more photons, its redox ability of photoinduced excitons is comparatively reduced. In contrast, for the PTBA sample, changing the electron acceptor can remarkably strengthen the reduction capacity, which is thermodynamically more favorable for achieving photocatalytic oxygen reduction reaction for the H_2_O_2_ production. By combining the results of E_CB_ with the optical E_g_ values, the valence band potentials (E_VB_) of PTBA and PTPA are determined as 1.31 and 1.75 V, respectively. Based on the above analysis, the band structures for the two photocatalysts are shown schematically in Figure 4b.

The separation or recombination of the photoinduced electron hole was monitored by steady-state photoluminescence (PL) spectra, as shown in Figure 4c. The lower PL intensity of PTBA indicates that the high crystallinity of PTBA can stimulate photogenerated charge separation and restrain the recombination of the electron hole. The internal electric field (IEF) is a driving force for charge separation and migration, which is a dynamic factor influencing photocatalytic performance. The relative IEF values of PTBA and PTPA can be obtained from the surface charge density and surface voltage following the reported method [33]. By simplifying the relative IEF as half power of the product of open circuit potential and surface charge, the IEF value of PTBA is 13.3 times greater than PTPA, according to the normalization of the calculated results (Figure 4d). The high crystallinity of PTBA effectively reduces intramolecular dipole cancellation, leading to a larger IEF, which could significantly promote photogenerated charge separation [34]. To further explore the importance of high crystallinity for promoting carrier separation and migration, the photocurrent (Figure 4e) and the electrochemical impedance spectroscopy (EIS) Nyquist plot (Figure 4f) measurements in a three-electrode cell system were carried out. Both I-t curves of PTBA and PTPA have correlated well with the on–off visible-light illumination, pointing to the photocatalytic activity of the samples. In addition, excellent stability of H_2_O_2_ production can be expected for the samples with good repeatability with the light off and on. The determined photocurrent densities of PTBA and PTPA are at about ~1.5 and ~0.2 μA cm^−2^, respectively. The stronger photocurrent response of PTBA proclaims to have a more efficient separation ability of photogenerated electron hole pairs, and the smaller EIS arc radius of PTBA indicates lower charge transfer resistance, as well as higher charge mobility.

### 2.4. Photocatalytic Performance and Mechanism Discussion

**Figure 5:**
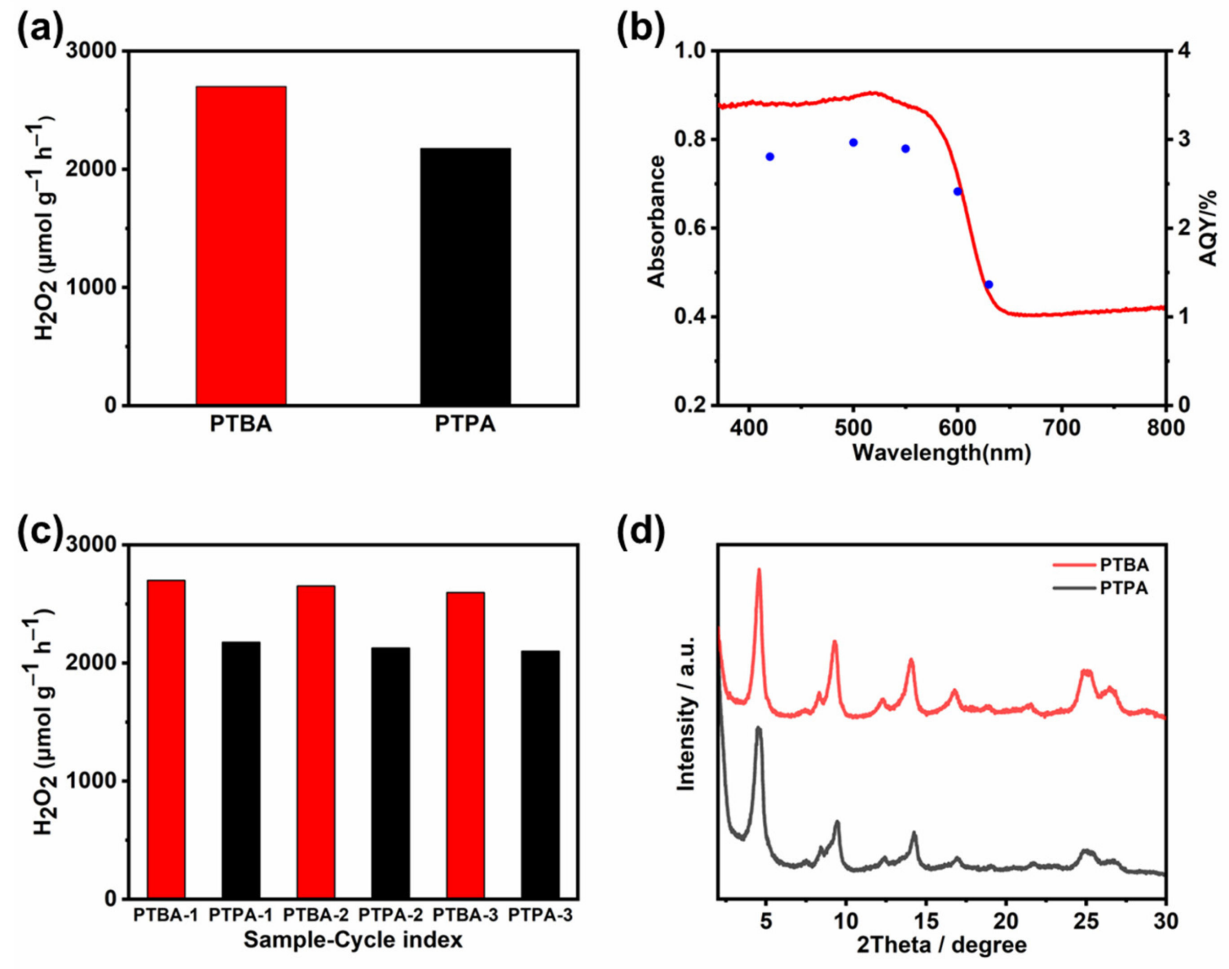
(**a**) The photocatalytic H_2_O_2_ evolution activities of PTBA and PTPA; (**b**) DRS spectrum of PTBA and the corresponding AQY for H_2_O_2_ generation; (**c**) cycling runs of photocatalytic H_2_O_2_ evolution of PTBA; (**d**) XRD patterns of PTBA before and after the reaction.

The photocatalytic activities of PTBA and PTPA toward H_2_O_2_ generation were determined under visible light irradiation with benzyl alcohol as the sacrificial reagent. No continuous O_2_ bubbling was adopted. As illustrated in Figure 5a, PTBA exhibits an excellent H_2_O_2_ yield of 2699 μmol g^−1^ h^−1^ under ambient test conditions, which is higher than PTPA (2176 μmol g^−1^ h^−1^). The enhanced photocatalytic activity of PTBA can be attributed to its high crystallinity. High crystallinity represents a directional arrangement of molecules, suppressing thermal vibration and reducing the possibility of exciton recombination, which leads to the promotion of separation and transfer of photocarriers [29,35]. Furthermore, the suitable electronic band structure allows for excellent reduction capacity, which is more favorable to photocatalytic from oxygen to H_2_O_2_. To understand the spectral distribution of the photocatalytic ability of PTBA, the apparent quantum yield (AQY) values were collected as a function of the wavelength of the incident light (Figure 5b). The AQY at a given wavelength was calculated from the following equation [36]:

$$\mathrm{AQY}\left(\%\right)=\frac{2\times {\mathrm{Number}\;\mathrm{of}\;\mathrm{evolved}\;H}_{2}O_{2}\;\mathrm{molecules}}{\mathrm{Number}\;\mathrm{of}\;\mathrm{incident}\;\mathrm{photons}}\times 100\%=\frac{2\times n\times N_{A}}{S\times P\times t\frac{\lambda }{h\times c}}\times 100\%$$

where n represents the number of evolved H_2_O_2_ molecules, N_A_ represents the Avogadro constant (6.02 × 10^23^ mol^−1^), S stands for the irradiation area, P is the light intensity (mW cm^−2^) determined by a calibrated power meter, t is the light irradiation time (s), λ represents the wavelength of the incident light, h is Planck constant (6.626 × 10^−34^ J s), and c is the light speed of 3 × 10^8^ m/s. At 420, 500, 550, 600, and 630 nm, PTBA renders AQY values of 2.81%, 2.96%, 2.89%, 2.41%, and 1.36%, respectively, which gives good agreement with the optical spectrum of PTBA, indicating that the photocatalytic reaction proceeds through light absorption. For PTBA, an average H_2_O_2_ generation of around 2.6 mmol g^−1^ h^−1^ was maintained after three cycles of photocatalytic reaction, which is a clear manifestation of high stability during photocatalysis (Figure 5c). No significant change is observable in the XPS signals of C 1s and O 1s after the photoreaction, suggesting that the chemical structure of PTBA is stable during the photocatalytic H_2_O_2_ evolution reaction (Figure S4). Also, the XRD pattern of PTBA after the photocatalytic cycling experiment is similar to the fresh sample, indicating its stability of morphology in photocatalytic reaction (Figure 5d).

**Figure 6:**
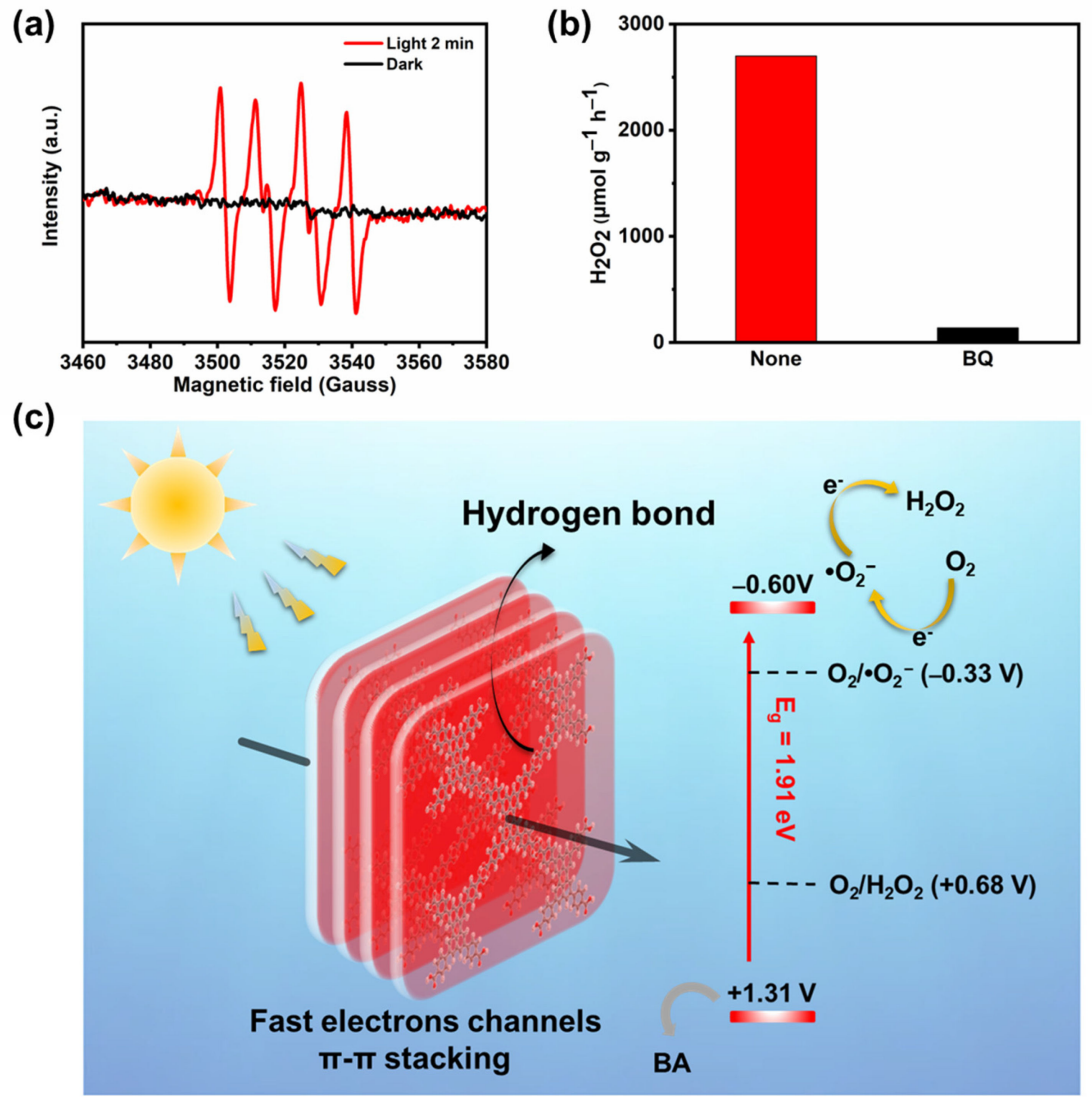
(**a**) EPR trapping experiments in the presence of DMPO as an electron-trapping agent; (**b**) comparison of photocatalytic H_2_O_2_ production by PTBA under the different conditions: control and BQ (0.1 mM); (**c**) schematic mechanism of H_2_O_2_ production for PTBA.

To investigate the photocatalytic H_2_O_2_ production pathway, electron paramagnetic spectroscopy (EPR) experiments were performed using 5,5-dimethyl-1-pyrroline N-oxide (DMPO) as the trapping agent. The typical signals of •O_2_^−^ are observed upon illumination in Figure 6a, demonstrating convincingly the two-step single-electron oxygen reduction reaction pathway to evolve H_2_O_2_ on the surface of PTPA [37]. The active intermediate was further verified with the addition of benzoquinone (BQ) as an •O_2_^−^ scavenger (Figure 6b). Consistent with the mechanism above, the formation rate of H_2_O_2_ was significantly suppressed in the presence of BQ, which confirms that •O_2_^−^ is the primary intermediate species during photocatalytic H_2_O_2_ generation.

Based on these results and discussions, a possible mechanism for photocatalytic H_2_O_2_ production by PTBA is proposed in Figure 6c. Under visible light irradiation, PTBA was excited by photons with sufficient energy to generate photogenerated charge carriers. Due to the directional arrangement of PTBA molecules by intermolecular hydrogen bonds, PTBA shows high crystallinity, leading to more electron hole pairs and effectively migrating to the catalyst surface. The experimental results of EPR and capture of the active species demonstrated that O_2_ was reduced by a photoexcited electron to produce •O^2−^, which quickly formed H_2_O_2_ by capturing another electron afterward. The sacrificial agents were oxidated by the photogenerated holes in the meantime.

## 3. Materials and Methods

### 3.1. Synthesis of MBP, PTBE, PTPE, PTBA, and PTPA

All the chemical reagents involved in the reactions were purchased from Shanghai Aladdin Biochemical Technology Co., Ltd. (Shanghai, China), Sun Chemical Technology (Shanghai) Co., Ltd. (Shanghai, China), Shanghai Macklin Biochemical Co., Ltd. (Shanghai, China), or Sinopharm Chemical Reagent, Co., Ltd. (Shanghai, China). All the reagents in the experiments were analytical grade and used as received without further purification.

2,5,8,11-tetrakis(4,4,5,5-tetramethyl-1,3,2-dioxaborolan-2-yl)perylene (MBP): Under Ar Perylene (499 mg, 1.98 mmol), 4,4,4′,4′,5,5,5′,5′-octamethyl-2,2′-bi(1,3,2-dioxaborolane) (2215 mg, 8.7 mmol), [Ir(OMe)COD]_2_ (65.6 mg, 0.1 mmol), and 4,4′-Dimethyl-2,2′-bipyridyl (54.4 mg, 0.2 mmol) were dispersed in 25 mL THF. The reaction mixture was degassed by three freeze-pump-thaw cycles and heated to 80 °C for 17 h. After completion, the reaction mixture was cooled down to RT and poured into 200 mL MeOH followed by filtration. Drying under a high vacuum overnight gave the product a yellow solid (1167 mg, 78%). ^1^H NMR (400 MHz, CDCl_3_): 8.62 (s, 4H, H_Ar_), 8.25 (s, 4H, H_Ar_), 1.43 (s, 48H, 48 × -CH_3_).

Tetra-tert-butyl 4,4′,4″,4‴-(perylene-2,5,8,11-tetrayl)tetrabenzoate (PTBE): Under Ar MBP (400 mg, 0.52 mmol), tert-butyl 4-iodobenzoate (724 mg, 2.36 mmol), [1,1′-bis(diphenylphosphine) Ferrocene] Palladium(II) chloride (II) (80 mg, 0.10 mmol), and potassium phosphate tribasic (1124 mg, 5.2 mmol) were dispersed in a mixture of 2 mL H_2_O and 22 mL DMF. The reaction mixture was degassed by three freeze-pump-thaw cycles and heated to 70 °C for 4 h. After completion, the reaction mixture was cooled down to RT followed by evaporation of organic solvents. The remaining solid was washed with water several times. Purification by column chromatography gave PTBE a yellow solid (384 mg, 76%). ^1^H NMR (400 MHz, CDCl_3_): 8.44 (s, 4H, H_Ar_), 8.12–8.06 (m, 8H, H_Ar_), 7.95 (s, 4H, H_Ar_), 7.80–7.76 (m, 8H, H_Ar_), 1.59 (s, 36H, 36 × -CH_3_). ^13^C NMR (151 MHz, CDCl_3_): 165.55, 145.20, 138.56, 135.14, 131.35, 131.22, 130.17, 127.61, 127.10, 126.78, 119.98, 81.23, 28.26, and 22.71. MS (MALDI TOF, *m*/*z*); exact mass calculated for C_64_H_60_O_8_ (M^+^): 956.2888; found: 956.2859.

4,4′,4″,4‴-(perylene-2,5,8,11-tetrayl)tetrabenzoic acid (PTBA): PTBE (306 mg, 0.32 mmol), NaOH (241.5 mg, 6.038 mmol), MeOH (32.0 mL), THF (32.0 mL), and H_2_O (3.2 mL) were added to a 150 mL bomb flask equipped with a stir bar. The resulting mixture was heated at 80 °C while stirring overnight. After completion, the mixture was cooled down to RT followed by evaporation of organic solvents. The remaining solid was dissolved in H_2_O and stirred at RT for an hour followed by filtration. The filtrate was acidified by HCl, and the precipitate was collected by centrifuge. Drying under a high vacuum overnight gave PTBA a red solid (270 mg, 90%). A total of 3 mL DMF containing 20 mg PTBA was added into 20 mL H_2_O and stirred for 5 min. Then, the mixture was poured into 17 mL ethanol. After stirring for 30 min, the precipitate was collected by centrifuge and dried at 50 °C overnight to obtain HOF PTBA with an ordered structure.

Octaisopropyl 4,4′,4″,4‴-(perylene-2,5,8,11-tetrayl)tetrakis(pyridine-2,6-dicarboxylate) (PTPE): Under Ar MBP (756.4 mg, 1 mmol), diisopropyl 4-iodopyridine-2,6-dicarboxylate (1696.5 mg, 4.5 mmol), [1,1′-bis(diphenylphosphine) Ferrocene] Palladium(II) chloride (II) (150 mg, 0.2 mmol), and potassium phosphate tribasic (2.1 g, 10 mmol) were dispersed in a mixture of 2 mL H_2_O and 22 mL DMF. The reaction mixture was degassed by three freeze-pump-thaw cycles and heated to 70 °C for 4 h. After completion, the reaction mixture was cooled down to RT followed by evaporation of organic solvents. The remaining solid was washed with water several times. Purification by column chromatography gave PTPE an orange solid (417 mg, 39%). ^1^H NMR (400 MHz, CDCl_3_): 8.67 (s, 4H, H_Ar_), 8.60 (s, 8H, 8 × -CH=CN), 8.18 (s, 4H, H_Ar_), 5.36–5.25 (p, 8H, 8 × -CH), 1.40 (d, *J* = 1.5 Hz, 48H, 48 × -CH_3_). ^13^C NMR (151 MHz, CDCl_3_): 164.14, 149.20, 148.84, 135.44, 130.79, 128.09, 127.33, 124.75, 119.84, 84.76, 69.54, 23.55, 19.84. MS (MALDI TOF, *m/z*); exact mass calculated for C_72_H_72_N_4_O_16_ (M^+^): 1248.3800; found: 1248.3287. 

4,4′,4″,4‴-(perylene-2,5,8,11-tetrayl)tetrakis(pyridine-2,6-dicarboxylic acid) (PTPA): PTPE (400 mg, 0.32 mmol), NaOH (241.5 mg, 6.038 mmol), MeOH (32.0 mL), THF (32.0 mL), and H_2_O (3.2 mL) were added to a 150 mL bomb flask equipped with a stir bar. The resulting mixture was heated at 80 °C while stirring overnight. After completion, the mixture was cooled down to RT followed by evaporation of organic solvents. The remaining solid was dissolved in H_2_O and stirred at RT for an hour followed by filtration. The filtrate was acidified by HCl, and the precipitate was collected by centrifuge. Drying under a high vacuum overnight gave PTPA a brown solid (270 mg, 90%). A total of 3 mL DMF containing 20 mg PTPA was added into 20 mL H_2_O and stirred for 5 min. Then, the mixture was poured into 17 mL ethanol. After stirring for 30 min, the precipitate was collected by centrifuge and dried at 50 °C overnight to obtain self-assembly PTPA. Due to its lack of solubility in conventional solvents (dichloromethane, chloroform, tetrahydrofuran, dimethyl sulfoxide, benzene, etc.), the structure of PTPA was characterized by FT-IR. Figure S1 shows the disappearance of an ester bond C=O stretch vibration at 1715 cm^−1^ in PTPE and the appearance of new peaks at approximately 1587 cm^−1^ in PTPA, which are assigned to the C=O of the carboxyl-terminal, confirming the accuracy of the structure of PTPA. 

### 3.2. Characterization of PTBA and PTPA

^1^H-NMR spectra were recorded on a Bruker 400 MHz instrument, and ^13^C-NMR spectra were recorded on a Bruker 400 MHz instrument. The chemical shifts were recorded in parts per million (ppm). Mass spectra were performed on Matrix-Assisted Laser Desorption/Ionization Time of Flight Mass Spectrometry. Fourier-transform infrared (FT-IR) spectra were recorded in transmission mode on a Nicolet Impact 410 spectrometer using KBr pellets in the range of 400–4000 cm^−1^. X-ray photoelectron spectroscopy (XPS) was detected with Al Kα as the excitation source on an ESCALAB 250 Xi spectrometer (Shimadzu/Krayos AXIS Ultra DLD). Powder X-ray diffraction (PXRD) data were recorded on a Bruker D8 Advance Powder X-ray Diffractometer using powder on a glass substrate, from 2θ = 2° up to 30° with a 0.01° increment. Transmission electron microscopy (TEM) was conducted on a Talos F200X TEM. Scanning electron microscopy (SEM) was conducted on a Helios G4 UC SEM-FIB (15 kV) equipped with an energy-dispersive spectrometer. The pore size, pore volume, and surface area of the samples were obtained by a Brunauer–Emmett–Teller (BET) instrument (Micromeritics ASAP 2460). DRS characterizations were obtained on a Varian Cary 500 spectrophotometer. Electron paramagnetic spectroscopy (EPR) was recorded on a Bruker EMXnano instrument. Electrochemical impedance spectroscopy (EIS), photocurrent, and Mott–Schottky plot measurements were conducted on a CHI650E electrochemical station with saturated calomel electrode (SCE), Pt wire, and glassy carbon electrode as the reference electrode, counter electrode, and working electrode, respectively. 

### 3.3. Photocatalytic H_2_O_2_ Production

A 5 mg catalyst was ultrasonically dispersed into 5 mL of benzyl alcohol as a sacrificial agent and 45 mL of deionized water. The suspension was illuminated by a 300 W Xe lamp with a 420 nm cutoff filter. All the reaction systems were kept at 15 °C and controlled by cooling water in the open-air atmosphere. A total of 3 mL of the suspension was withdrawn at given time intervals followed by centrifugation to remove the photocatalysts. The concentrations of H_2_O_2_ evolved were detected using iodometric assays. The photocatalyst, after removing benzyl alcohol and water, was used for the cycle test. Furthermore, AQY measurements for photocatalytic H_2_O_2_ production were performed under monochromatic light irradiation (300 W xenon lamp, λ = 420, 500, 550, 600, and 630 nm) with 10% filter film. 

## 4. Conclusions

In summary, a hydrogen-bonded organic framework PTBA and an amorphous analog photocatalyst PTPA were successfully synthesized and applied to photocatalytic H_2_O_2_ generation. Remarkably, the H_2_O_2_ production rate of PTBA can reach as high as 2699 μmol g^−1^ h^−1^ under visible light irradiation, which is over 500 μmol g^−1^ h^−1^ higher than PTPA. The enhanced photocatalytic performance of PTBA is attributed to the high crystallinity confirmed by the sharp diffraction peak of PXRD analysis and the lattice fringes in TEM images. The ordered arrangement of PTBA molecules reduces the structural defects that play the role of trapping photogenerated carriers, which leads to the promotion of exciton separation and transfer. The experimental results of the EPR and capture of the active species indicate that the photocatalyst evolves H_2_O_2_ primarily through the two-step single-electron oxygen reduction reaction pathway. Our research brings insights to the design of high-crystalline materials with expected performance in photocatalytic H_2_O_2_ generation.

## Data Availability

The data in this study are available from the corresponding author upon reasonable request.

## Supplementary Materials

The following supporting information can be downloaded at https://www.mdpi.com/article/10.3390/molecules28196850/s1, Figure S1: XPS spectra of PTPA; Figure S2: Tauc plots of PTBA and PTPA; Figure S3: Mott–Schottky plots of PTBA and PTPA; Figure S4: XPS spectra of PTBA before and after reaction; Figures S5–S10: NMR and MS (MALDI TOF) spectra.

## Sample Availability

Samples of some compounds may be available from the authors.
