# Supplementary material for: A High Crystalline Perylene-Based Hydrogen-Bonded Organic Framework for Enhanced Photocatalytic H2O2 Evolution
Source: Molecules. 2023 Sep 28;28(19):6850. doi: 10.3390/molecules28196850 (PMC10574088; doi:10.3390/molecules28196850)
Supplement: Supplementary file 1 [file molecules-28-06850-s001.zip › molecules-2613289-supplementary.pdf]

Electronic Supplementary Information (ESI)

A High Crystalline Perylene-based Hydrogen-bonded Organic Framework for Enhanced Photocatalytic H_2O_2 Evolution

Mengke Hu †, Chenxi Wu †, Shufan Feng and Jianli Hua *

Key Laboratory for Advanced Materials, School of Chemistry and Molecular Engineering, East China University of Science and Technology, 130 Meilong Road, Shanghai 200237, China; y30210401@mail.ecust.edu.cn (M.H.); y30230414@mail.ecust.edu.cn (C.W.); y12223040@mail.ecust.edu.cn (S.F.)

* Correspondence: jlhua@ecust.edu.cn

† These authors contributed equally to this work.

Table of Contents

Section	Title	Pages
Figure S1	XPS spectra of **PTPA**	S3
Figure S2	Tauc plots of **PTBA** and **PTPA**	S3
Figure S3	Mott-Schottky plots of **PTBA** and **PTPA**	S3
Figure S4	XPS spectra of **PTBA** before and after reaction	S4
Figure S5-10	NMR and MS (MALDI TOF) spectra	S5-7

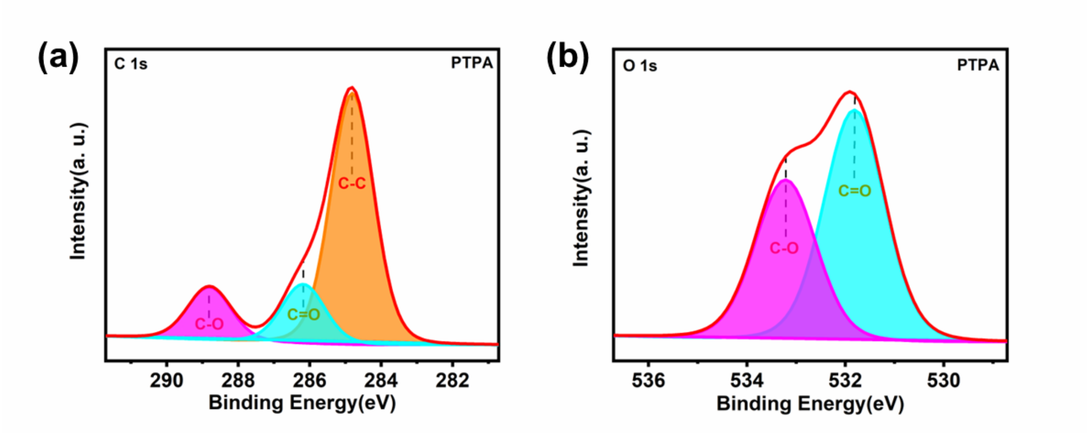

Figure S1. XPS spectra of **PTPA** (a) C 1s and (b) O 1s.

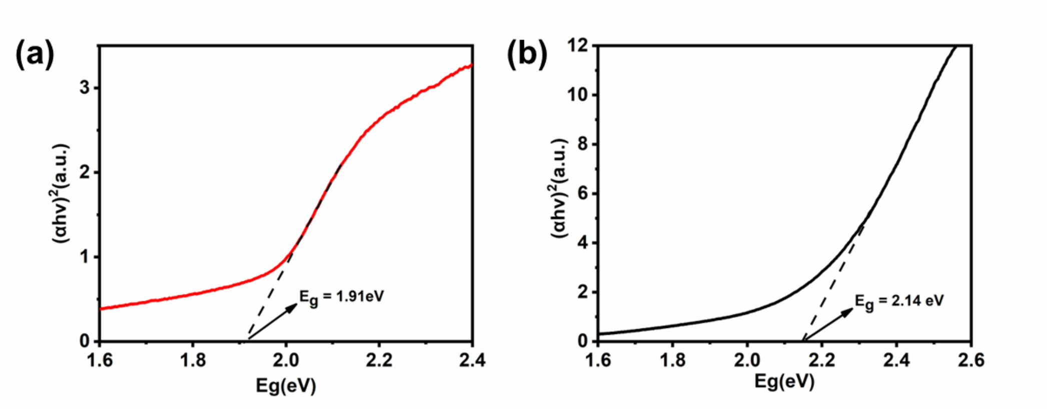

Figure S2. Tauc plots of (a) **PTBA** and (b) **PTPA**.

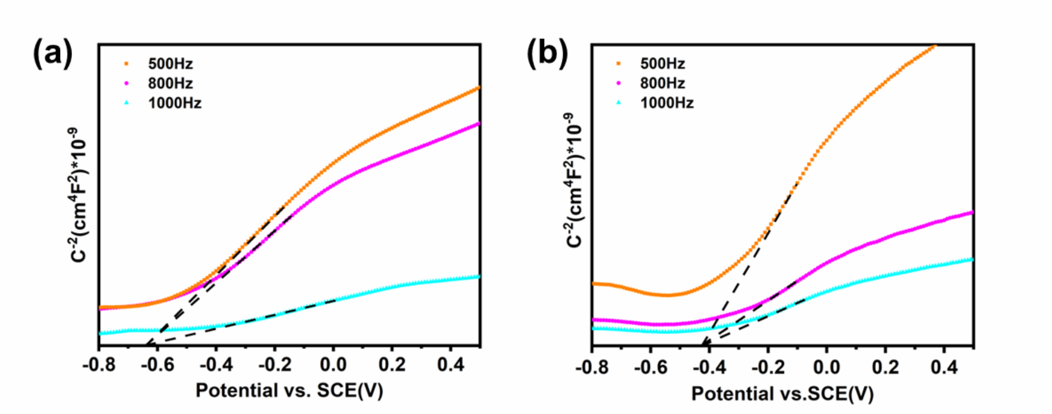

Figure S3. Mott-Schottky plots of (a) **PTBA** and (b) **PTPA**.

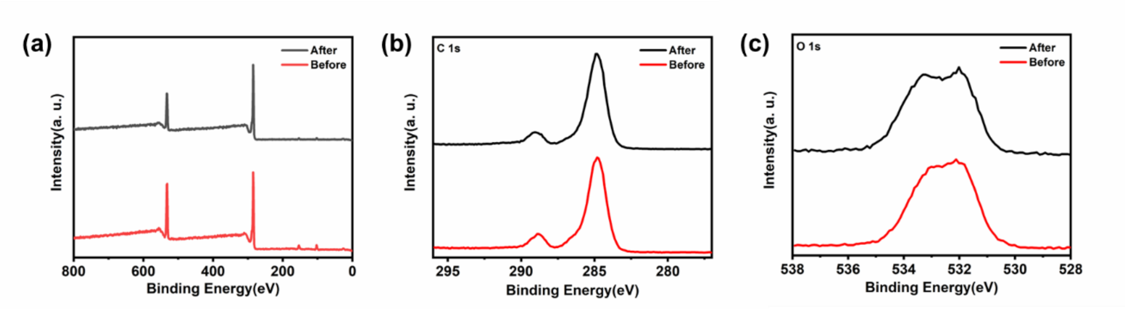

Figure S4. XPS spectra of **PTBA** before and after reaction (a) survey, (b) C 1s, and (c) O 1s.

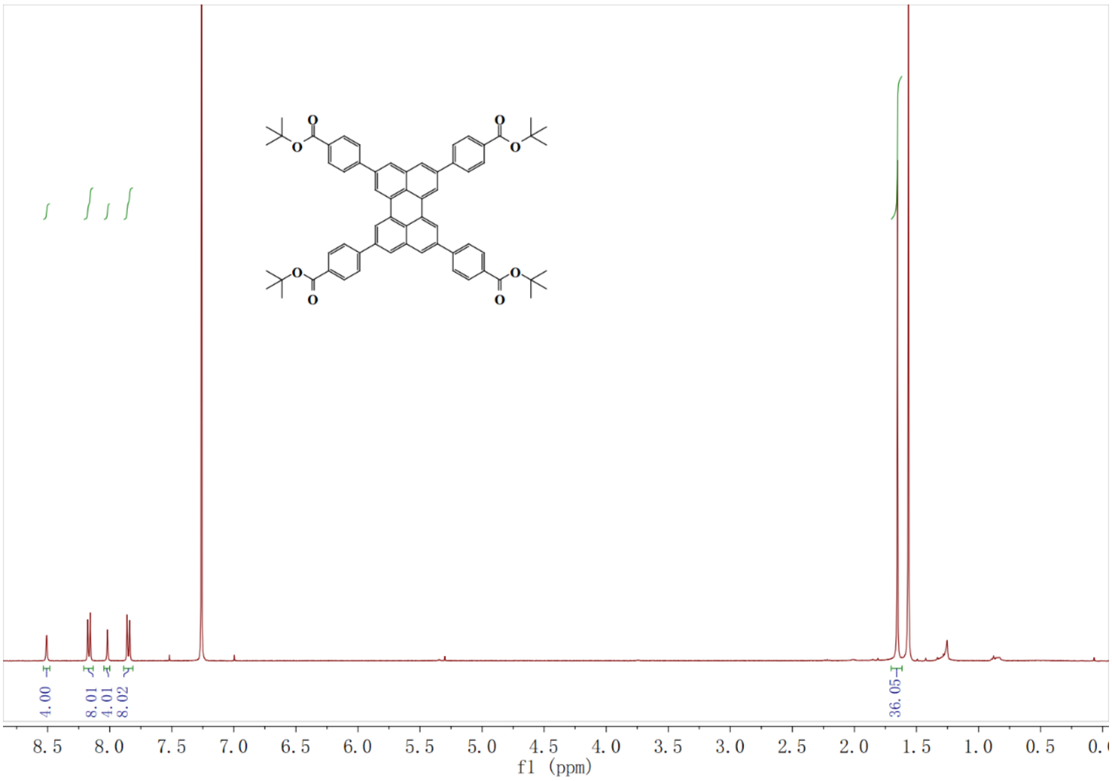

Figure S5. ^1H NMR spectrum of **PTBE** in $CDCl_3$.

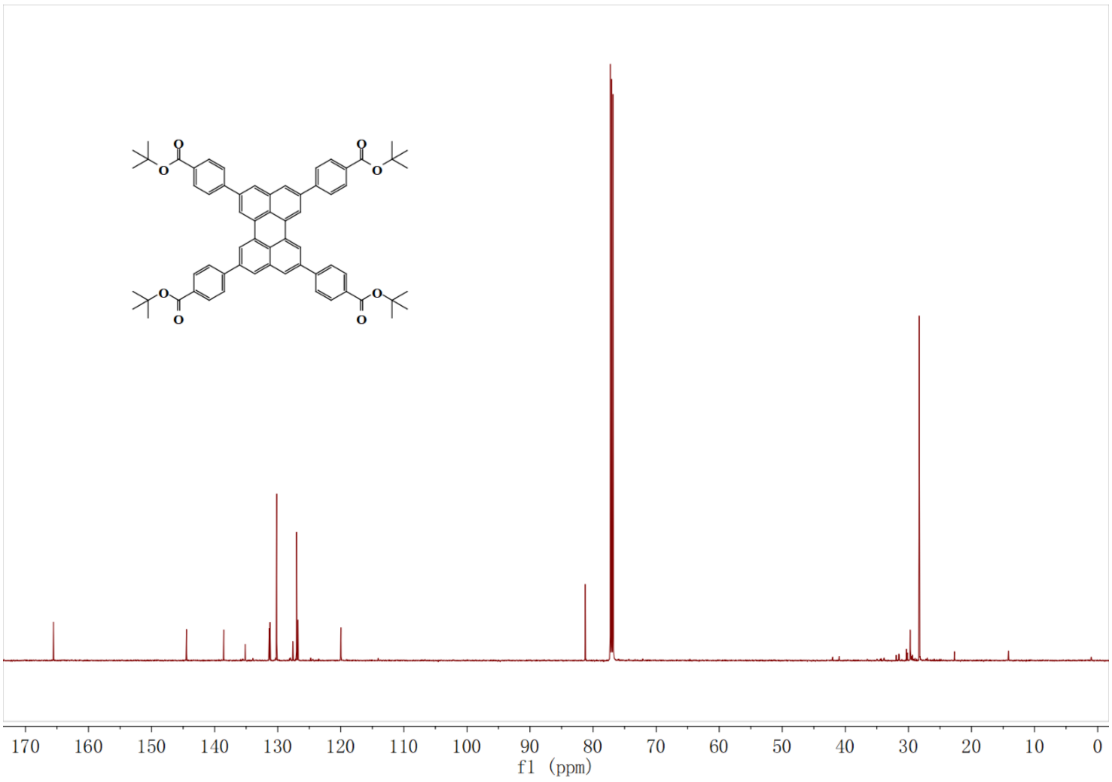

Figure S6. ^{13}C NMR spectrum of **PTBE** in $CDCl_3$.

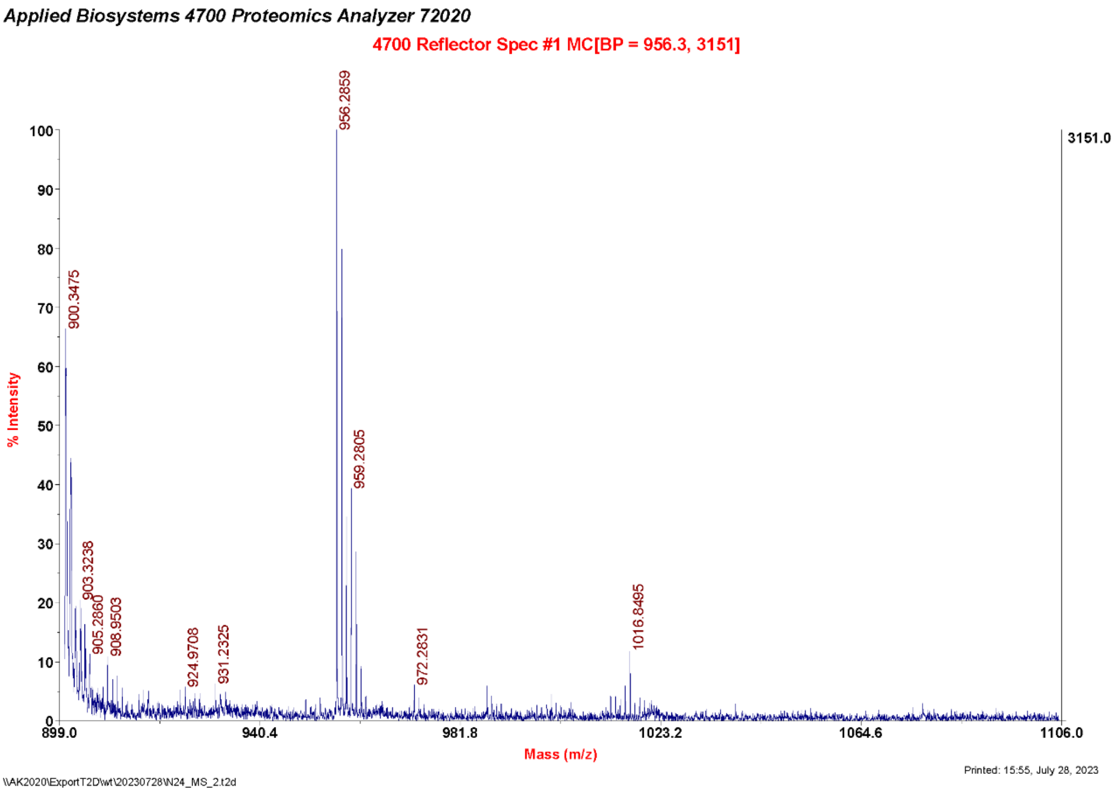

Figure S7. MS (MALDI TOF) of **PTBE**.

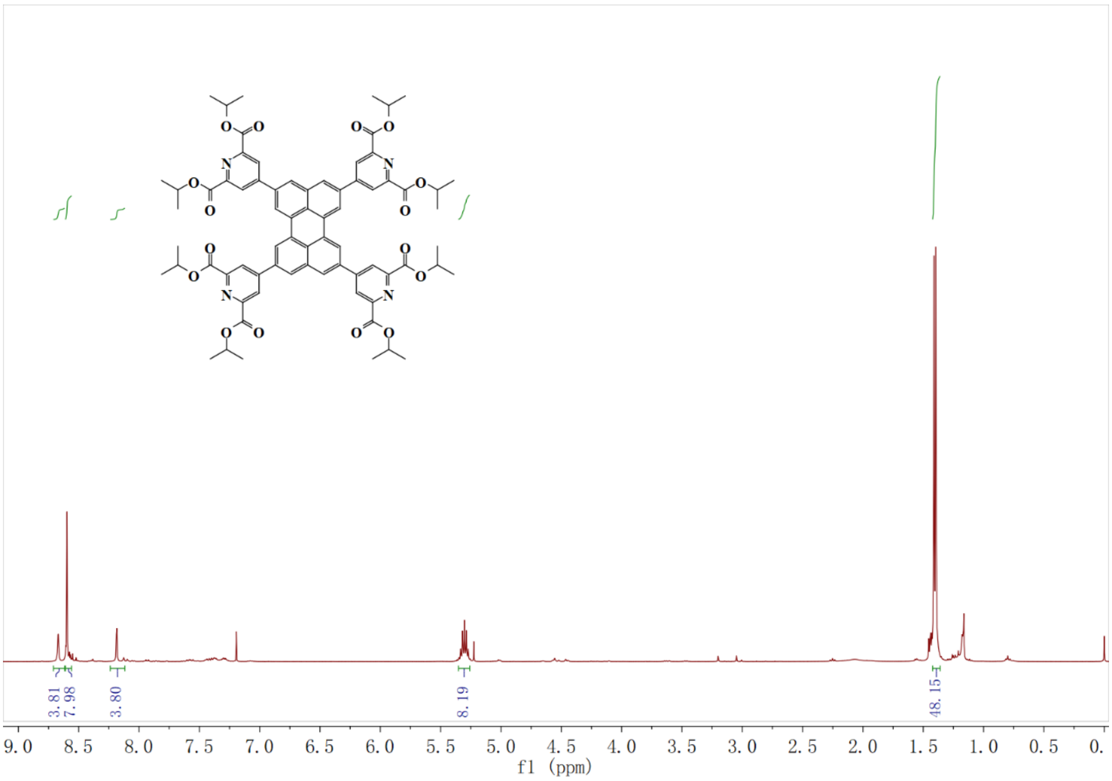

Figure S8. ^{1}H NMR spectrum of **PTPE** in $CDCl_3$.

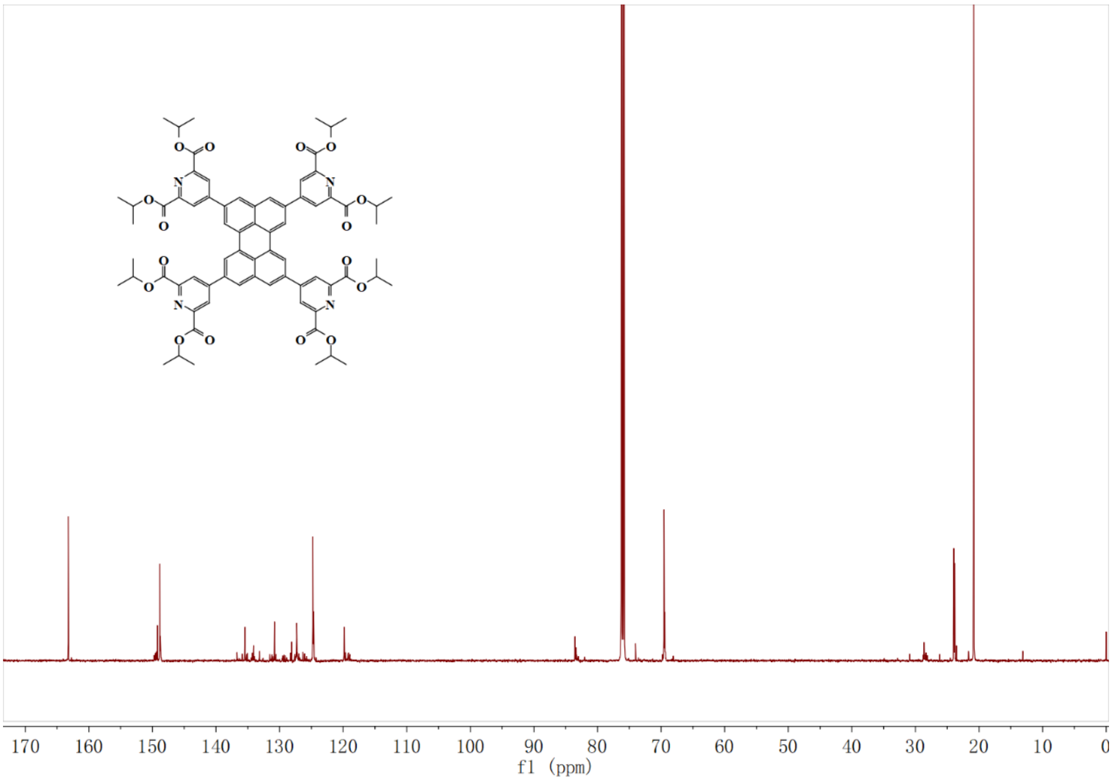

Figure S9. ^{13}C NMR spectrum of **PTPE** in $CDCl_3$.

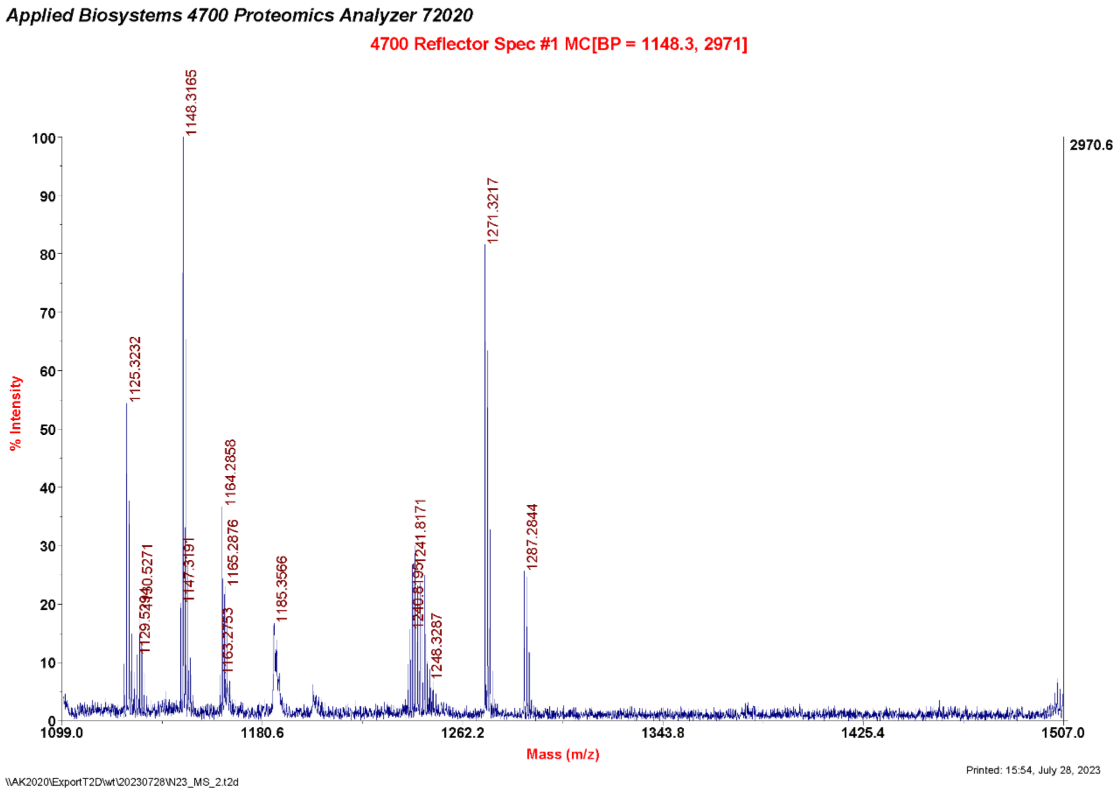

Figure S10. MS (MALDI TOF) of **PTPE**.
